# Tuberculosis retreatment ‘others’ in comparison with classical retreatment cases; a retrospective cohort review

**DOI:** 10.1186/s12889-015-2195-2

**Published:** 2015-09-02

**Authors:** Mary G. Nabukenya-Mudiope, Herman Joseph Kawuma, Miranda Brouwer, Peter Mudiope, Anna Vassall

**Affiliations:** Track Tuberculosis Activity Project-Management Sciences for Health, Plot no. 15, Princess Anne Drive Bugolobi, P.O. Box 71419, Kampala, Uganda; German Leprosy and Tuberculosis Relief Association, Kampala, Uganda; Public Health and Tuberculosis Consultancy, Tilburg, The Netherlands; Uganda AIDS Commission, Kampala, Uganda; London School of Hygiene and Tropical Medicine, London, United Kingdom

## Abstract

**Background:**

Many of the countries in sub-Saharan Africa are still largely dependent on microscopy as the mainstay for diagnosis of tuberculosis (TB) including patients with previous history of TB treatment. The available guidance in management of TB retreatment cases is focused on bacteriologically confirmed TB retreatment cases leaving out those classified as retreatment ‘others’. Retreatment ‘others’ refer to all TB cases who were previously treated but with unknown outcome of that previous treatment or who have returned to treatment with bacteriologically negative pulmonary or extra-pulmonary TB. This study was conducted in 11 regional referral hospitals (RRHs) serving high burden TB districts in Uganda to determine the profile and treatment success of TB retreatment ‘others’ in comparison with the classical retreatment cases.

**Methods:**

A retrospective cohort review of routinely collected National TB and Leprosy Program (NTLP) facility data from 1 January to 31 December 2010. This study uses the term classical retreatment cases to refer to a combined group of bacteriologically confirmed relapse, return after failure and return after loss to follow-up cases as a distinct group from retreatment ‘others’. Distribution of categorical characteristics were compared using Chi-squared test for difference between proportions. The log likelihood ratio test was used to assess the independent contribution of type of retreatment, human immunodeficiency virus (HIV) status, age group and sex to the models.

**Results:**

Of the 6244 TB cases registered at the study sites, 733 (11.7 %) were retreatment cases. Retreatment ‘others’ constituted 45.5 % of retreatment cases. Co-infection with HIV was higher among retreatment ‘others’ (70.9 %) than classical retreatment cases (53.5 %). Treatment was successful in 410 (56.2 %) retreatment cases. Retreatment ‘others’ were associated with reduced odds of success (AOR = 0.44, 95 % CI 0.22,0.88) compared to classical cases. Lost to follow up was the commonest adverse outcome (38 % of adverse outcomes) in all retreatment cases. Type of retreatment case, HIV status, and age were independently associated with treatment success.

**Conclusion:**

TB retreatment ‘others’ constitute a significant proportion of retreatment cases, with higher HIV prevalence and worse treatment success. There is need to review the diagnosis and management of retreatment ‘others’.

## Background

The World Health Organization (WHO) treatment guidelines recommend that all previously-treated TB patients should be managed according the TB retreatment category, while their sputum is cultured and tested for drug susceptibility (DST) [1]. However, few countries have the required laboratory capacity to improve access to DST services to all TB retreatment patients. Therefore, many countries remain unclear on the best management of TB retreatment cases. Of particular concern is the category of TB patients classified as retreatment ‘others’. These refer to all TB cases, previously treated but with unknown outcome of that previous treatment or who return for treatment with bacteriologically negative pulmonary or extra-pulmonary TB. This study uses the term classical retreatment cases to refer to all bacteriologically confirmed relapse, return after failure and return after lost to follow-up (LTFU) cases as a distinct group from retreatment ‘others’.

Uganda has limited capacity to conduct culture and DST investigations in TB retreatment patients. A study conducted in three regional referral hospitals (RRHs) in Uganda showed that only 13 % of 114 registered relapse smear-positive or treatment after failure cases had their sputum samples sent to National TB Reference Laboratory for culture and DST [2]. Since 2002, Uganda has notified an increasing number of TB retreatment cases from 1500 to about 4000 cases per year [3]. Of the 47,650 total TB cases Uganda notified to the WHO in 2013, 4028 (8.5 %) were TB retreatment cases [4]. TB retreatment ‘others’ constituted a third of the total retreatment cases notified in 2012 [3].

An important step in understanding how to manage retreatment ‘others’ is to better understand their outcomes. Previous studies in other settings have observed different treatment outcomes, HIV status and management approaches between classical TB retreatment cases and retreatment ‘others’ [5–7]. A study in India found that retreatment ‘others’ significantly had better treatment outcomes than classical retreatment cases [7]. Another study in Zimbabwe found that retreatment ‘others’ constituted 40 % of recurrent TB with no difference in treatment outcomes by HIV status [6]. 65 % of retreatment cases in Malawi were retreatment ‘others’ with over half of them treated with standard TB regimen for new cases [5]. This study seeks to add this emerging evidence base on how this group of patients differs by setting, to answer the following research question: what is the profile and treatment success of TB retreatment ‘others’ compared to the classical retreatment cases in Uganda?

## Methods

A retrospective hospital-based review of routinely collected TB data on TB retreatment patients started on TB treatment from 1st January to 31st December 2010. The data were extracted between May and June 2012.

### Study setting

This study was conducted in 11 RRHs of Uganda serving mostly districts with high TB burden. In 2009, it was observed that districts with RRHs notified an average of 114 retreatment cases each compared to an average of 32 retreatment cases notified by districts without RRHs (unpublished NTLP reports). The study thus systematically selected 11 high burden RRHs based on the burden of TB. The study sites were: Arua, Fort-Portal, Gulu, Hoima, Jinja, Kabale, Lira, Masaka, Mbarara, Mbale and Soroti RRHs.

### Case definitions and treatment of retreatment TB patients

In Uganda, a TB retreatment case is defined as a person previously treated with anti-TB drugs for a month or more and is being treated again, in line with WHO definitions [1, 8]. The retreatment category is further classified either as ‘relapse’, ‘treatment after failure’, ‘return after LTFU’ or ‘others’. Relapses are patients who become bacteriologically positive after having been treated for TB and declared cured or treatment completed. Treatment after failure are patients who, while on first line anti-TB treatment are bacteriologically positive at 5 months or later during the course of treatment. Return after LTFU patients are those who return to treatment and are bacteriologically positive after having interrupted treatment for more than 2 months. Retreatment ‘others’ refer to all TB cases that do not fit the above definitions such as patients with history of TB treatment for a month or more but with no bacteriological confirmation of TB for the current episode.

In line with WHO definitions, Ugandan NTLP classifies treatment outcomes as; cured, treatment completed, treatment failure, died, LTFU and transferred-out. Treatment success refers to a combination of cured and treatment completed. In this study, adverse outcome refers to a combination of LTFU, died, treatment failure and transferred-out.

The retreatment regimen in Uganda consists of two (2) months streptomycin (S), rifampicin (R), isoniazid (H), pyrazinamide (Z), and ethambutol (E). This is followed by one (1) month RHZE and five (5) months RHE. The retreatment regimen(2SRHZE/1RHZE/5RHE) is recommended for all bacteriologically positive TB retreatment cases [1, 8]. Both NTLP and WHO guidelines are silent on the management of TB retreatment ‘others’ in settings with limited TB DST capacity. In Uganda, it is at the discretion of the clinician to decide the TB treatment regimen to use in the management of retreatment ‘others’. At the time of the study, routine culture and DST for retreatment cases had been rolled out to the study sites with varying levels of implementation [2].

### Study variables, source of information and data collection

Records of routinely collected variables within the hospitals’ unit TB registers that were analyzed included: patient demographic (age and sex); clinical (disease classification, pre-treatment smear status and HIV status); treatment-related (type of retreatment and treatment regimen) characteristics and treatment outcomes. In Uganda, each TB patient is registered in the unit TB register by the health facility staff at the start of treatment and individual patient records updated at every visit during the course of treatment. The district TB and Leprosy supervisor (DTLS) enters TB patients registered on treatment from all TB diagnostic and treatment health facilities within that particular district into the district TB register. Information on patients that transferred to other facilities within the same district is captured by the DTLS and conveyed back to the registering facility. More information on patients transfers between districts in the same zone is exchanged during quarterly zonal performance reviews attended by DTLSs before compiling quarterly district TB and Leprosy reports on notification and treatment outcomes. At the time of the study, the reporting unit at the NTLP central unit was the district. Using an anonymous standardized data collection tool, study variables were extracted from the hospital’ TB unit registers by one trained research assistant and all entries were verified by the first author. The respective district TB registers were used to ascertain definitive patient treatment outcomes that were missing in the unit registers.

### Data entry and analysis

Data was entered into EpiData version 3.1 (The EpiData Association, Odense, Denmark)and analyzed in STATA version 11.2 (Stata Corp, College Station, TX, USA).

HIV status was categorized into positive, negative and unknown. Age was categorized using cut offs that made meaningful differences between the categories. Descriptive analysis of patient characteristics was computed. Distribution of patient characteristics by type of retreatment cases (classical vs. retreatment ‘others’) was computed. The differences in distribution of categorical characteristics were compared using Chi-square test for difference between proportions at a significance level of *P*-value equal to 0.05.

Treatment outcome was analyzed as a binary variable of success versus all other outcomes. Odds ratio was the measure of association. Logistic regression was used to identify patient characteristics that were independently associated with treatment success. Characteristics that had *P*-value equal or less than 0.05 at bivariate level were assessed further in a multivariate model. In the multivariate analysis, characteristics that were not significant at *p*-value equal or less than 0.05 were dropped. The multivariable model was determined using forward regression with a two-sided *P*-value equal or less than 0.05. Sex was included as a priori in the final model. The log likelihood ratio test was used to assess the independent contribution of explanatory variables to the models.

### Ethical approval

As this study was a review of routinely collected NTLP data at RRHs, approval was obtained from Ministry of Health and Joint Clinical Research Centre Institutional Review Board as the local ethical body. The protocol was also approved by London School of Hygiene Tropical Medicine ethics review committee.

## Results

**Fig. 1:**
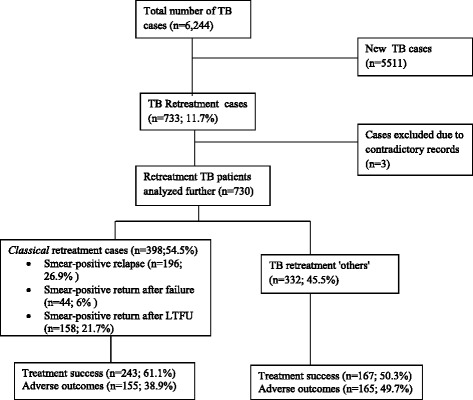
Proportion of TB retreatment cases and outcomes at eleven regional referral hospitals, Uganda

Of the 6244 TB cases registered at the 11 RRHs, 733 (11.7 %) were retreatment cases (Fig. 1). Three retreatment cases were excluded from subsequent analyzes due to contradictory records. Table 1 shows that majority of retreatment cases were males (71.6 %) and in age group 15–44 years (70 %). Overall, 690 (94.5 %) retreatment cases had a documented HIV test result.

**Table 1:** Frequency of retreatment TB patients’ characteristics and their distribution by type of retreatment cases registered at the eleven RRHs, 2010 (*n* = 730 cases)

Characteristic	All retreatment cases	Type of retreatment cases		*P*-value*
		Classical TB retreatment cases;	TB retreatment ‘others’;	
	n (%)	*n* = 398 (%)	*n* = 332 (%)	
Sex				
Male	523 (71.6)	308 (77.4)	215 (64.8)	<0.001
Female	207 (28.4)	90 (22.6)	117 (35.2)	
Age (years)^a^				
<15	39 (5.4)	3 (0.8)	36 (10.8)	<0.001
15-44	509 (69.8)	293 (73.8)	216 (65.1)	
>44	181 (24.8)	101 (25.4)	80 (24.1)	
Anatomical site				
Pulmonary	689 (94.4)	398 (100.0)	291 (87.7)	<0.001
Extrapulmonary	41 (5.6)	0 (0.0)	41 (12.3)	
Disease classification				
Sputum smear-positive	400 (54.8)	398 (100.0)	2 (0.6)	<0.001
Sputum smear-negative	267 (36.6)	0 (0.0)	267 (80.4)	
No smear done	22 (3.0)	0 (0.0)	22 (6.6)	
Extrapulmonary	41 (5.6)	0 (0.0)	41 (12.4)	
HIV status				
Negative	266 (36.4)	174 (43.7)	92 (27.7)	<0.001
Positive	424 (58.1)	200 (50.3)	224 (67.5)	
Unknown	40 (5.5)	24 (6.0)	16 (4.8)	
Retreatment sub-category				
Sputum smear-positive relapse	196 (26.9)	196 (49.3)	0 (0.0)	<0.001
Sputum smear-positive failure	44 (6.0)	44 (11.1)	0 (0.0)	
Sputum smear-positive return after LTFU	158 (21.6)	158 (39.7)	0 (0.0)	
Retreatment ‘others’	332 (45.5)	0 (0.0)	332 (100.0)	
Treatment regimen				
Retreatment: 2SRHZE/1RHZE/5RHE	582 (79.7)	378 (95.0)	204 (61.5)	<0.001
New: 2RHZE/6EH or 2RHZ/4RH	116 (15.9)	19 (4.8)	97 (29.2)	
Other regimen^b^	32 (4.4)	1 (0.2)	31 (9.3)	

*P-values are from Pearson’s chi-squared test or Fisher’s exact test for the difference in the distribution of the categorical characteristics across the types of retreatment cases

^a^1 patient had missing data

^b^Other regimen included: 3RHZE/5RHE = 13; 2SRHZ/4-12RH = 14; 2SRHZE/6EH = 2; 3SRH/6RH = 1; unknown = 2

Retreatment ‘others’ constituted 45.5 % of retreatment cases. Like the classical retreatment cases, retreatment ‘others’ were mostly males (65 %), and in the age group 15–44 years (65 %). Significantly, lesser (62 %) of retreatment ‘others’ were treated with the standard retreatment regimen (2SRHZE/1RHZE/5RHE) compared to 95 % of classical retreatment cases. About a third of the retreatment ‘others’ were treated with the standard regimen for new TB patients.

Table 2 shows that HIV prevalence was higher among retreatment ‘others’ (70.9 %) than classical retreatment cases (53.5 %). HIV co-infection was 61.5 % among 690 retreatment patients that had a documented HIV test result. Females had a higher HIV prevalence (70.7 %) than males (57.8 %). Of the 424 patients with an HIV-positive test result, 385 (91 %) and 221 (52 %) were provided with Cotrimoxazole preventive therapy (CPT) and antiretroviral therapy (ART) respectively.

**Table 2:** Prevalence of HIV by patient characteristics, retreatment type among retreatment TB patients with known HIV test results at eleven RRHs, Uganda (*n* = 690)

Characteristic	Total		Classical retreatment cases		‘Others’ retreatment cases		*P*-value*
		HIV Positive		HIV Positive		HIV Positive	
		*n* (%)		*n* (%)		*n* (%)	
Overall	690	424^b^ (61.4)	374	200 (53.5)	316	224 (70.9)	<0.001**
Sex							
Male	492	284 (57.8)	289	145 (50.2)	203	139 (68.5)	0.022
Female	198	140 (70.7)	85	55 (64.7)	113	85 (75.2)	
Age group, years^a^							
<15	35	24 (68.6)	3	2 (66.7)	32	22 (68.8)	<0.001
15-44	488	316 (64.8)	277	157 (56.7)	211	159 (75.4)	
>44	166	83 (50.0)	94	40 (42.6)	73	43 (58.9)	
Anatomical site							
Pulmonary	650	391 (60.2)	374	200 (53.5)	276	191 (69.2)	<0.001
Extrapulmonary	40	33 (82.5)	0	0 (0.0)	40	33 (82.5)	
Treatment regimen							
Retreatment: 2SRHZE/1RHZE/5RHE	553	329 (59.5)	357	192 (53.8)	196	137 (69.9)	<0.001
New:2RHZE/6EH or 2RHZ/4RH	106	73 (68.9)	16	8 (50.0)	90	65 (72.2)	
Other regimen	31	22 (71.0)	1	0 (0.0)	30	22 (73.3)	

*P-values are from either Pearson’s chi-squared test of Fischer’s exact tests for the difference between given characteristics and the type of retreatment among only HIV positive patients

**P-value from Z-test for two proportions

^a^1 patient had missing data on this variable

^b^Cotrimoxazole preventive therapy and antiretroviral treatment were documented among 385 (91 %) and 221 (52 %) of all HIV positive TB retreatment patients respectively

Table 3 shows that treatment was successful in 410 (56.2 %) of the 730 retreatment cases. Adverse outcomes were; 16.4 % LTFU, 9.9 % died, 2.6 % failed on treatment while 5.1 % transferred-out and 9.9 % were not-evaluated. Table 4 shows that retreatment ‘others’ were associated with reduced odds of treatment success [odds ratio (OR) =0.65, 95 % CI 0.48, 0.87] compared to the classical retreatment cases. Anatomical site and treatment regimen were not associated with treatment success. Using multivariable analysis, odds of treatment success remained lower among retreatment ‘others’ compared to the classical retreatment cases after adjusting for age group, HIV status and sex (Adjusted OR (AOR)) = 0.60, 95% CI 0.44, 0.82). Unknown HIV status was significantly associated with lower odds of treatment success compared to known HIV status (AOR = 0.44, 95% CI 0.22, 0.88). Together with type of retreatment case, age group (less than 15 years) became significantly associated with treatment success (AOR = 2.32, 95% CI 1.12, 4.81).

**Table 3:** Outcomes of retreatment cases by WHO retreatment category

Type of retreatment	Treatment outcome		Adverse outcomes				
	Successful	Adverse	Failure	Died	LTFU	Transfer-out	Not -evaluated
	*n* (%)	*n* (%)	*n* (%)	*n* (%)	*n* (%)	*n* (%)	*n* (%)
Smear-positive relapse: *n* = 196	134 (68.4)	62 (31.6)	9 (4.6)	9 (4.6)	23 (11.7)	7 (3.6)	14 (7.0)
Smear-positive return after failure: *n* = 44	30 (68.2)	14 (31.8)	4 (9.1)	3 (6.9)	5 (11.4)	0 (0.0)	2 (4.5)
Smear-positive return after LTFU: *n* = 158	79 (50)	79 (50.0)	3 (1.9)	19 (12)	26 (16.4)	12 (7.6)	19 (12.0)
Retreatment ‘others’: *n* = 332	167 (50.3)	165 (49.7)	3 (0.9)	41 (12.3)	66 (19.9)	18 (5.4)	37 (11.0)
Total; *n* = 730	410 (56.2)	320 (43.8)	19 (2.6)	72 (9.9)	120 (16.4)	37 (5)	72 (9.9)

*P* < 0.001 for the difference between the type of retreatment and treatment outcome using Pearson’s chi-squared test

**Table 4:** Patient characteristics associated with binary treatment success among TB retreatment cases registered in eleven RRHs of Uganda (*n* = 730)

Characteristics	Total	Treatment Success n (%)	Unadjusted OR (95 % CI)	*P*-value*	Adjusted^b^ OR (95 % CI)	*P*-value*
Overall	730	410 (56.2)				
Type of retreatment case						
*Classica*l retreatment cases	398	243 (61.1)	1.00		1.00	
Retreatment ‘others’	332	167 (50.3)	0.65 (0.48, 0.87)	0.004**	0.60 (0.44, 0.82)	0.001**
HIV status						
Positive	424	236 (55.7)	1.00		1.00	
Negative	266	159 (59.8)	1.18 (0.87, 1.62)	0.288	1.13 (0.82, 1.56)	0.452
Unknown	40	15 (37.5)	0.48 (0.24, 0.93)	0.030**	0.44 (0.22, 0.88)	0.020**
Age group^a^						
15-44	509	292 (57.4)	1.00		1.00	
<15	39	27 (69.2)	1.67 (0.83, 3.37)	0.143	2.32 (1.12, 4.81)	0.024**
≥45	181	90 (49.7)	0.73 (0.52, 1.03)	0.073	0.75 (0.53, 1.06)	0.102
Sex						
Male	523	296 (56.6)	1.00		1.00	
Female	207	114 (55.1)	0.94 (0.68, 1.30)	0.708	0.97 (0.69, 1.35)	0.844
Anatomical site						
Pulmonary	689	388 (56.3)	1.00			
Extrapulmonary	41	22 (53.7)	0.90 (0.48, 1.69)	0.739		
Treatment regimen						
Retreatment: 2SRHZE/1RHZE/5RHE	582	322 (55.3)	1.00			
New: 2RHZE/6EH or 2RHZ/4RH	116	66 (56.9)	1.07 (0.71, 1.59)	0.756		
Other regimen	32	22 (68.8)	1.78 (0.83, 3.82)	0.137		

*Wald P-value

**Significant at *P* = 0.05

^a^1 patient had missing data

^b^Adjusted for HIV status, age, and sex

## Discussion

Retreatment ‘others’ constitute almost half of the retreatment cases in the RRHs of Uganda. Compared to the classical retreatment cases, more cases of retreatment ‘others’ were HIV positive. And more than a third of retreatment ‘others’ were not managed with the standard retreatment regimen. Fewer (half) retreatment ‘others’ succeeded on treatment (50.3 %) compared to six in ten of the classical retreatment cases (61.1 %). Lost to follow up was the commonest adverse outcome for both retreatment groups.

Nearly half of the retreatment cases in this study were retreatment ‘others’ compared to one in three cases notified nationally [3]. Probably, RRHs receive mostly very sick patients whose sputum is likely to test negative on Ziehl Neelsen (ZN) smear test due to their inability to mount an immune response and/ or produce sufficient sputum for microscopy. In addition, the TB diagnosis in RRHs is likely to be made by relatively highly qualified clinicians with capacity and/or bias to rely on their clinical acumen to diagnose TB even in the absence of a positive AFB sputum result. The presence of high TB-HIV co-infection rates in our study may account for the observed high proportion of retreatment ‘others’ [9]. The proportion of retreatment ‘others’ in this study is comparable to those from Zimbabwe and India [6, 7], but less than the proportion observed from a study conducted in a large registration centre of Malawi [5].

Overall, treatment success was low at 50 % in retreatment ‘others’ and 61 % in classical retreatment cases, compared to 71 % treatment success notified to WHO [3]. This study considered patients in referral hospitals who may be different from other TB patients from lower levels of care on a number of factors. Due to the referral cascade, patients with atypical forms of TB or drug-resistant TB are likely to be managed at RRHs and hence likely to exhibit poor treatment outcomes. However, the observed factors like the weakness in recording and reporting system, inability to track these patients (15 % of participants didn’t have definitive outcomes) and the low uptake of ART (52 %) among HIV co-infected patients may also explain the low treatment success. The 52 % ART uptake observed in the study population was higher than the national average of 24 % [10]. The difference between treatment success in this study and that reported to WHO may be because we evaluated all retreatment cases registered and not only classical retreatment cases that are routinely evaluated. Nonetheless, the results support previous findings from a smaller study conducted in three RRHs in Uganda [2].

The outcomes for retreatment ‘others’ in this study were worse than those of retreatment ‘others’ reported in India as compared to the classical retreatment cases [7]. This difference across settings may be influenced by factors such as difference in treatment regimens’ [5], drug resistance [11], co-morbidity [12], delay in diagnosis or even misdiagnosis [5, 13], adherence levels or having another pulmonary or extra-pulmonary disease for which they are not adequately treated.

Similar to the results reported in previous studies [5, 6], this study found that a higher proportions of retreatment ‘others’ were co-infected with HIV compared to the classical retreatment cases. Just like in other studies, we found that patients’ knowledge of their HIV status is beneficial [9, 14, 15]. 90 % of study participants that were found to be HIV-positive were started on CPT and half of them started on ART as well. The high uptake of HIV testing coupled with good initiation of CPT in this study could have resulted in observing no difference in treatment success between HIV-positive and negative patients. Age group and unknown HIV status were only significant independent predictors, but did not modify the effect of type of TB retreatment case on treatment outcome.

The findings of this study should be viewed with the following limitations. Firstly, this was a retrospective study utilizing hospital TB registers which could be prone to inaccuracies resulting from poor recording and completeness in the patient data and compromise the validity of the finding. However, this study found that 94.5 % of smear-positive retreatment cases had smear result correctly recorded. In addition, completeness of treatment outcomes was improved by use of the district TB registers whereby 28 % (176/621) of definitive outcomes among study participants were obtained.

We could not establish treatment outcomes in 15 % of the study participants even after reviewing district TB registers. There were no differences in patient characteristics between those who had complete information on the outcomes and those who had missing outcomes. However, the high proportion of missing treatment outcome could have introduced bias in determining treatment success or underreported deaths or LTFU among the study participants.

This study highlights the importance of ensuring appropriate management for retreatment ‘others’ given the relatively poor outcomes. A first key step is to ensure that this high number of retreatment ‘others’ is not a result of misdiagnosing drug-resistant TB or a false positive diagnosis of TB. More accurate TB diagnostic tools like the GeneXpert MTB/RIF that are currently available in all the study sites may have a role in providing access to a confirmation of TB in this special group.

Secondly, the continued notification of high proportions of retreatment ‘others’ to national authorities and Global TB Program calls for clear guidance on the management of retreatment ‘others’ including further definition of treatment regimen(s) in high HIV prevalence settings and limited TB diagnostic capacity. A future prospective study involving culture and drug-susceptibility testing could be conducted in programmatic settings to further understand the appropriate treatment regimens in such patients.

Thirdly, the observed high LTFU in the retreatment patients especially the retreatment ‘others’ calls for focused and innovative interventions to ensure treatment adherence in this group of patients. Social incentives and community outreach may have a role to play in reducing the loss to follow-up of these groups.

Fourthly, high HIV prevalence among retreatment cases especially the TB retreatment ‘others’ calls for better strategies of improving provision of the full range of TB/HIV collaborative services to reduce the burden of HIV in this group of patients.

Finally, further research is recommended at different levels of the TB treatment program to further clarify the importance of patient, health worker and system related factors on treatment success among retreatment cases to complement the findings of this study; and design the most appropriate response to ensure favorable outcomes from this underserved and evaluated group of TB patients.

## Abbreviations

AFB: Acid fast bacilli
AOR: Adjusted odds ratio
ART: Antiretroviral therapy
CI: Confidence interval
CPT: Cotrimoxazole preventive therapy
DST: Drug susceptibility testing
DTLS: District TB and Leprosy supervisor
E: Ethambutol
H: Isoniazid
HIV: Human immunodeficiency virus
LFTU: Lost to follow up
LSHTM: London School Of Hygiene and Tropical Medicine
MTB/RIF: Mycobacterium tuberculosis and rifampicin-resistance mutations
NTLP: National Tuberculosis and Leprosy Programme
OR: Odds ratio
R: Rifampicin
RRHs: Regional referral hospitals
S: Streptomycin
TB: Tuberculosis
WHO: World Health Organization
Z: Pyrazinamide
ZN: Ziehl Neelsen
